# Exome-wide analysis identify multiple variations in olfactory receptor genes (*OR12D2* and *OR5V1*) associated with autism spectrum disorder in Saudi females

**DOI:** 10.3389/fmed.2023.1051039

**Published:** 2023-02-01

**Authors:** Noor B. Almandil, Maram Adnan Alismail, Hind Saleh Alsuwat, Abdulla AlSulaiman, Sayed AbdulAzeez, J. Francis Borgio

**Affiliations:** ^1^Department of Clinical Pharmacy Research, Institute for Research and Medical Consultations (IRMC), Imam Abdulrahman Bin Faisal University, Dammam, Saudi Arabia; ^2^Department of Genetic Research, Institute for Research and Medical Consultations (IRMC), Imam Abdulrahman Bin Faisal University, Dammam, Saudi Arabia; ^3^College of Medicine, Imam Abdulrahman Bin Faisal University, Dammam, Saudi Arabia; ^4^Department of Neurology, College of Medicine, Imam Abdulrahman Bin Faisal University, Dammam, Saudi Arabia

**Keywords:** autism spectrum disorder, Saudi females, coding variants, single nucleotide polymorphism, haplotyping

## Abstract

**Background:**

Autism Spectrum Disorder (ASD) is a multifactorial, neurodevelopmental disorder, characterized by deficits in communication, restricted and repetitive behaviors. ASD is highly heritable in Saudi Arabia; indecencies of affected individuals are increasing.

**Objectives:**

To identify the most significant genes and SNPs associated with the increased risk of ASD in Saudi females to give an insight for early diagnosis.

**Methods:**

Pilot case–control study mostly emphasized on the significant SNPs and haplotypes contributing to Saudi females with ASD patients (*n* = 22) compared to controls (*n* = 51) without ASD. With the use of allelic association analysis tools, 243,345 SNPs were studied systematically and classified according to their significant association. The significant SNPs and their genes were selected for further investigation for mapping of ASD candidate causal variants and functional impact.

**Results:**

In females, five risk SNPs at *p* ≤ 2.32 × 10^−05^ was identified in association with autism. The most significant exonic variants at chromosome 6p22.1 with olfactory receptor genes (*OR12D2* and *OR5V1*) clustered with high linkage disequilibrium through haplotyping analysis. Comparison between highly associated genes (56 genes) of male and female autistic patients with female autistic samples revealed that 39 genes are unique biomarkers for Saudi females with ASD.

**Conclusion:**

Multiple variations in olfactory receptor genes (*OR5V1* and *OR12D2*) and single variations on *SPHK1, PLCL2, AKAP9* and *LOC107984893* genes are contributing to ASD in females of Arab origin. Accumulation of these multiple predisposed coding SNPs can increase the possibility of developing ASD in Saudi females.

## 1. Introduction

Autism Spectrum Disorder (ASD) is a range of neurodevelopmental and neuropsychiatric disorders that start appearing from early childhood and lasts throughout the person’s life (1, 2). Autism is among the most heritable and severe form of ASD (3), characterized by deficits in communication as well as repetitive and restricted behaviors as reported in *the Diagnostic and Statistical Manual, Fifth Edition (DSM-5)* (4). The World Health Organization (WHO) revealed statistics of 1 in 160 children to be diagnosed with ASD (5). In 2017, the latest ASD statistics in Saudi Arabia revealed that one per 167 individual is affected by autism (6). ASD is commonly multifactorial and many studies suggested interactions between immunological, neurological, environmental and genetic factors (7, 8). Tremendous sex bias of ASD shows that males are more affected than females with a male to female ratio of 3:1 (9). Several studies investigated the genetic risk factors against ASD in females, yet the key factors remain unknown (10–12). This paper identifies some genetic variables susceptible to cause autism in Saudi females, addresses the correlation between some diseases and pathways and genetic variants in Saudi females.

## 2. Methodology

### 2.1. Sample collection

The present study is conducted in accordance with the Declaration of Helsinki and received approval from the Institutional Review Board (IRB) of Imam Abdulrahman Bin Faisal University (IRB-2016-13-152). Out of 73 female age matched samples were included, 22 were cases and 51 were controls (Table 1). The present study sheds light on potential genetic contributors to autism in Saudi female subjects. Buccal cell samples were collected from the study subjects upon receiving the signed informed consent. All the samples were collected from the King Fahad Hospital of the University, Al Khobar, Saudi Arabia.

**Table 1:** Characteristics of Saudi female patients with autism and controls without autism.

Parameter	Control group *n* = 51	Case group *n* = 22	Value of *p*
Age (year)	7.73 ± 3.13	7.09 ± 3.93	0.2251
Weight (kg)	30.01 ± 13.61	25.11 ± 11.92	0.1034
Height (cm)	121.38 ± 23.33	119.2 ± 20.69	0.3707
Body mass index	19.95 ± 4.97	16.70 ± 2.03	0.0033^*^

The data are presented as the mean values ± standard deviations. ^*^Significant at *p* ≤ 0.05.

### 2.2. DNA extraction and genotyping

To extract DNA from buccal cell samples, the Gentra Puregene Buccal Cell Kit (Qiagen, Hilden, Germany) was used. The buccal cells were collected by scraping the inside of the mouth 10 times with given sterile brush. DNA was extracted within 3 h from the collection, 300 μl Cell lysis solution was dispensed in a 1.5 ml tube, incubated at 65°C for 15 min, and 1.5 μl of proteinase K was added and incubated at 55°C for 60 min. Then we added 100 μl protein precipitation reagent and incubated for 5 min on ice and centrifuged (13,000–16,000 ×*g* for 3 min). Supernatant was mixed with 300 μl isopropanol and 0.5 μl glycogen, centrifuged for 5 min at 13,000–16,000 ×*g*. The supernatant was discarded, the DNA pellet was washed with 70% ethanol and suspended the DNA in TE buffer. The Human Exome Bead Chip Kit v1.0 and v1.1 Illumina (San Diego, CA, United States), which is constituted of 243,345 putative functional exonic markers, was used with Illumina iScan for the microarray genotyping. DNA processing was performed in accordance with the manufacturer’s protocol and all genotyping data were obtained from iScan control software (Illumina). DNA extraction and microarray genotyping and analysis took place in the genetics research laboratory of the Institute for Research and Medical Consultation, Imam Abdulrahman Bin Faial University, Dammam, Saudi Arabia. The procedures were executed between 2016 and 2019. The Infinium HTS workflow is a rapid 3 days work flow, in brief: The PicoGreen dsDNA quantification reagent was used to quantify double-stranded DNA samples. The quantified DNA samples were processed in 96 well plates. The quantified DNA samples were denatured and neutralized to prepare them for amplification. All the DNA samples were incubated uniformly to amplify, to generate a sufficient quantity of each individual DNA sample to be used in the Infinium HTS Assay. We Incubated the MSA3 plate with amplified DNA in the Illumina hybridization oven for 20–24 h at 37°C. Then to fragment the DNA, an endpoint fragmentation was used. A 100% 2-propanol and precipitation reagents were used to precipitate the DNA. Then, re-suspended the precipitated and fragmented DNA. The re-suspended DNA was dispensed onto bead chips and incubated for hybridization of each DNA sample to specific section of the bead chip. Afterwards, the bead chips were prepared for the staining process. Then the un-hybridized and non-specifically hybridized DNA samples were washed from the bead chips, added labeled nucleotides to extend primers hybridized to the sample, and stains the primers. For imaging the bead chip we followed the instructions in the System Guide for instrument to scan. Intensity files from iScan of the individual DNA samples from the exome chip were to perform the genotyping. Sample sheets with sample information, such as plate ID, cell ID, gender and so on were used for fetching the data from intensity files to perform the genotyping using GenomeStudio 2,0 software (Illumina.

### 2.3. Statistical and functional analysis

Initial quality check of call rate was fulfilled using GenomeStudio 2,0 software (Illumina). Only one control was eliminated from the analysis due to a call rate of < 0.98% and remaining samples were re-clustered. Using the Chi-square test with 1 degree of freedom (df), Hardy–Weinberg equilibrium (HWE) was tested individually for all the variants. Reference SNP ID numbers and gene names were acquired from SNP-Nexus (13) and Kaviar (14). To assess the outcomes of different alleles and haplotypes, 95% confidence interval, odds ratios and case–control association analyses were calculated using gPlink version 2.050 (15) and Haploview version 4.2 (16). The *p* values < 0.001 were regarded as significant. DAVID 6.7 (17) and Enricher (18) were utilized to annotate the highly significant remarkable (*p* < 1 × 10^−05^) genes for functional implications.

## 3. Results

**Figure 1:**
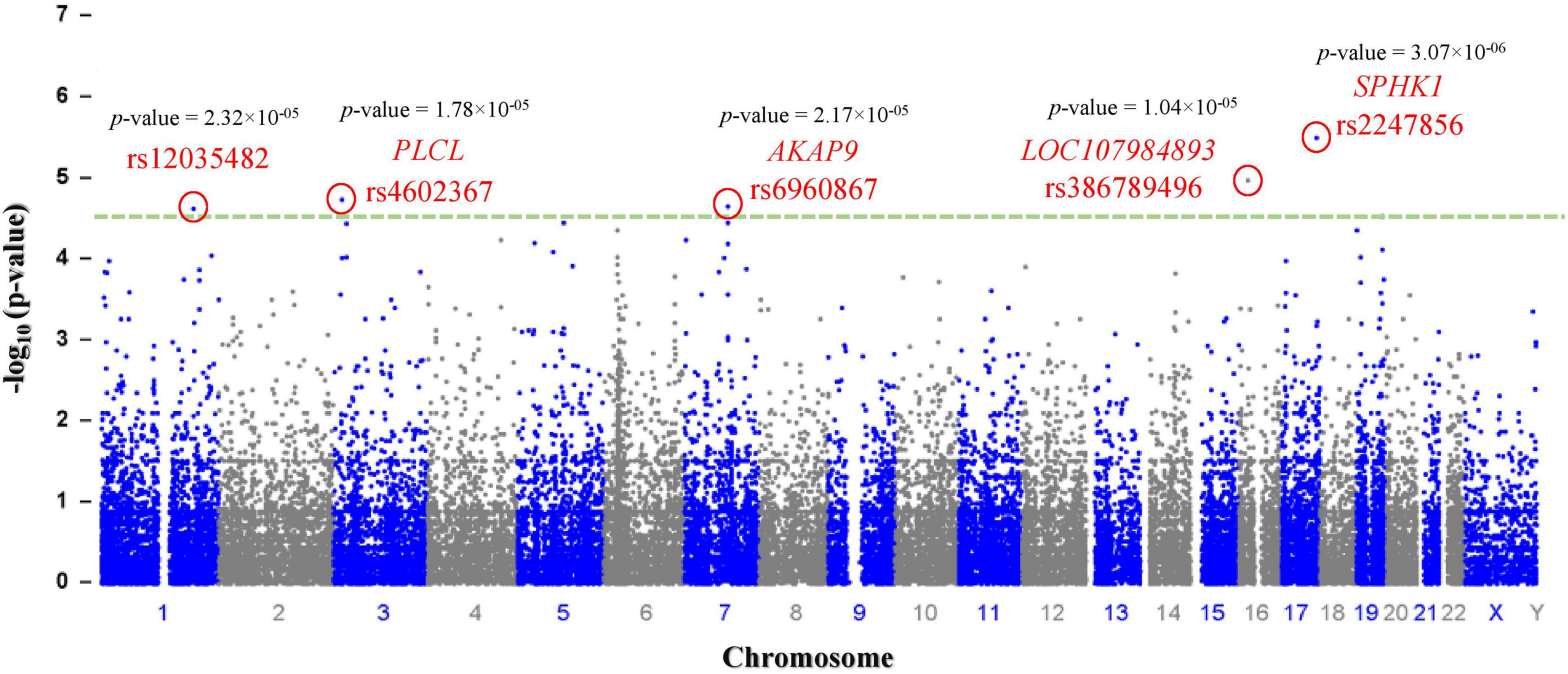
Manhattan plot: a total of (*n* = 243,345) SNPs are plotted according to *p*-values (*y*-axis) and their position in the genome (*x*-axis). The most significant candidate nucleotide variants rs2247856 (*SPHK1*), rs386789496 (*LOC107984893*), rs4602367 (*PLCL*), rs6960867 (*AKAP9*) and rs12035482 on chromosome 17, 16, 3, 7and 1 respectively, exceed the significance threshold line (*p* = 1.00 × 10^–4.5^ - green dash line) indicating a statistically significant correlation with autism.

Genotyping (Illumina) data were submitted to the NCBI (National Center for Biotechnology Information) Gene Expression Omnibus (GEO) repository [GEO accession number: GSE221098; BioProject accession numbers: PRJNA912746; GEO accession numbers for individual samples: GSM6845201-GSM6845273].^1^ After filtering 243,345 SNPs according to their *p* values, 280 SNPs with *p* < 0.0001 were selected as significant (*p* < 9.44 × 10^−05^; Table 2). The most significant SNPs suggesting a correlation with autism were rs2247856 (*p* = 3.069 × 10^−06^ at *SPHK1*), rs386789496 (*p* = 1.036 × 10^−05^ at *LOC107984893*), rs4602367 (*p* = 1.783 × 10^−05^ at *PLCL2*), rs6960867 (*p* = 2.17 × 10^−05^ at *AKAP9*) and rs12035482 (*p* = 2.32 × 10^−05^) located on chromosome 17, 16, 3, 7 and 1, respectively, (Figure 1). All the significant SNPs of Saudi females autistic patients with *p* < 0.00018 are listed in Supplementary Table 1 in which all obey the hardy–Weinberg equilibrium.

**Table 2:** The most significant SNPs associated with autism in Saudi females.

S.NO	CHR	SNP ID	BP	MA	MAF	Gene	AA	Value of *p*	CHISQ	OR (L95-U95)	Case, control frequencies	HWpval
1	17	rs2247856	74,381,555	A	0.247	*SPHK1*	A	3.07 × 10^−06^	21.77	6.28(2.77–14.23)	0.500, 0.137	0.0017
2	16	rs386789496	17,988,303	A	0.473	*LOC107984893*	A	1.04 × 10^−05^	19.44	5.5(2.48–12.17)	0.750, 0.353	0.0117
3	3	rs4602367	17,053,499	A	0.336	*PLCL2*	A	1.78 × 10^−05^	18.41	4.96(2.32–10.6)	0.591, 0.225	0.2088
4	7	rs6960867	91,712,698	G	0.397	*AKAP9*	G	2.17 × 10^−05^	18.03	4.86(2.28–10.38)	0.659, 0.284	0.588
5	1	rs12035482	195,738,953	A	0.493	none	G	2.32 × 10^−05^	17.91	0.18(0.08–0.42)	0.773, 0.390	0.0717
6	19	rs7507442	53,278,953	G	0.486	*ZNF600*	G	2.83 × 10^−05^	17.53	5.05(2.28–11.15)	0.750, 0.373	0.0396
7	7	rs6964587	91,630,620	A	0.403	*AKAP9*	T	3.43 × 10^−05^	17.17	4.8(2.22–10.37)	0.667, 0.294	0.3397
8	5	rs160632	96,503,523	G	0.445	*RIOK2*	C	3.46 × 10^−05^	17.15	4.76(2.21–10.27)	0.705, 0.333	0.1332
9	3	rs9854207	27,614,316	C	0.363	none	C	3.53 × 10^−05^	17.11	4.64(2.18–9.85)	0.614, 0.255	0.1288
10	19	rs142920057	334,472	C	0.121	*MIER2*	G	4.29 × 10^−05^	16.74	8.143(2.64–25.09)	0.300, 0.050	0.6628
11	6	rs2073149	29,365,423	A	0.493	*OR5V1*	A	4.30 × 10^−05^	16.74	4.89(2.21–10.82)	0.750, 0.380	0.3153
12	4	rs1339	154,631,563	G	0.197	*RNF175*	C	5.60 × 10^−05^	16.23	5.50(2.28–13.25)	0.405, 0.110	0.5161
13	7	rs10488360	4,411,209	A	0.452	none	A	5.67 × 10^−05^	16.21	4.56(2.12–9.81)	0.705, 0.343	0.4184
14	5	rs409045	34,628,627	G	0.37	none	C	6.14 × 10^−05^	16.06	4.41(2.08–9.33)	0.614, 0.265	0.4098
15	7	rs1063243	91,726,927	C	0.411	*AKAP9*	C	6.27 × 10^−05^	16.02	4.42(2.08–9.39)	0.659, 0.304	0.5296
16	19	rs57088011	53,454,387	G	0.062	*ZNF816*	C	7.33 × 10^−05^	15.72	22.44(2.71–185.8)	0.182, 0.010	0.4609
17	5	rs11556045	73,985,215	G	0.233	*HEXB*	A	7.95 × 10^−05^	15.57	0.048(0.00–0.36)	0.977, 0.676	0.282
18	1	rs669408	232,519,150	C	0.35	none	C	8.77 × 10^−05^	15.38	4.5(2.06–9.79)	0.600, 0.250	1
19	3	rs2642926	27,615,419	A	0.459	none	T	9.15 × 10^−05^	15.3	4.37(2.03–9.38)	0.705, 0.353	0.0125
20	19	rs7248104	7,224,431	A	0.459	*INSR*	A	9.15 × 10^−05^	15.3	4.37(2.03–9.38)	0.705, 0.353	0.9049
21	6	rs2073153	29,364,835	C	0.472	*OR12D2*	T	9.17 × 10^−05^	15.3	0.21(0.09–0.47)	0.773, 0.418	0.3848
22	3	rs17272796	17,077,268	G	0.336	*PLCL2*	C	9.29 × 10^−05^	15.28	4.27(2.01–9.06)	0.568, 0.235	0.2088
23	7	rs10260011	84,709,356	A	0.226	*SEMA3D*	T	9.44 × 10^−05^	15.25	4.77(2.10–10.86)	0.432, 0.137	0.5474

S.NO: serial number; CHR: chromosome; SNP ID: single nucleotide polymorphism ID; BP: base pair position at the respective chromosome as per GRCh37.p13; MA: minor allele name; MAF: frequency of minor allele in controls; AA: associated allele; *p*: value of *p*; ChisQ: basic allelic test Chi-square; *p*: value of p; OR: odd ratio; L95: lower bound of 95% confidence interval for odds ratio; U95: upper bound of 95% confidence interval for odds ratio.; CCF: case, control frequencies; HWpval: value of *p* of Hardy–Weinberg equilibrium.

**Figure 2:**
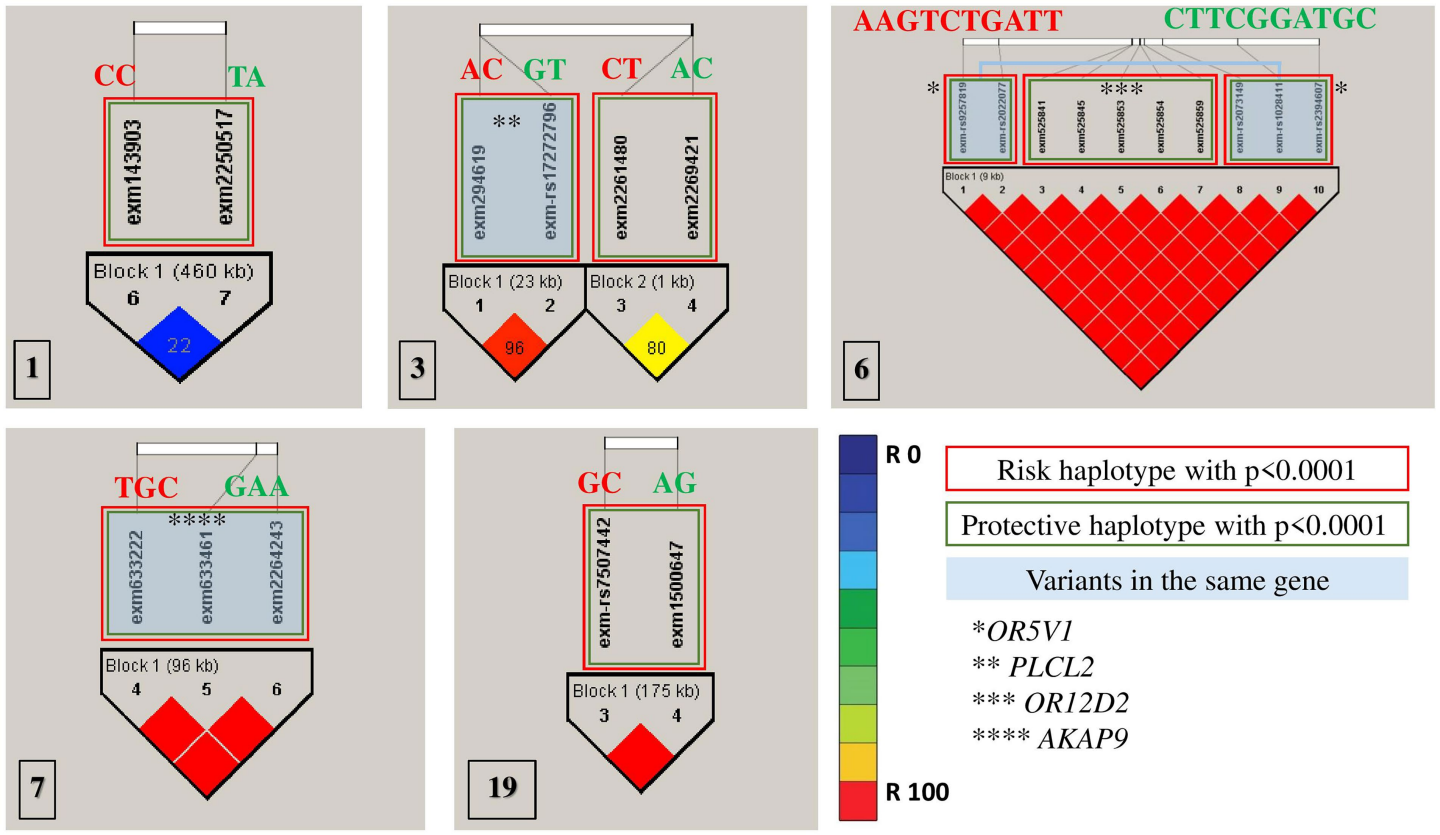
Haplotype blocks representing the linkage disequilibrium of chosen SNPs on chromosomes of autistic females in Saudi Arabia. The numbers at the bottom left of each picture correspond to the chromosome number. * refers to the genes’ names. Red rectangles are the most risk haplotypes, green rectangles are the most protective haplotypes and light blue rectangles highlights the SNPs which are located in the same gene. Further details are in Table 3.

All association tests were screened using minor alleles’ frequency in controls, value of *p* of Hardy–Weinberg equilibrium and type 1 error rate to achieve the strongest genetic predisposition and imputed for linkage disequilibrium in HapMap SNPs in multiple chromosomes (Figure 2). The haplotype analysis implemented on SNPs with significance of *p* < 0.0001 were classified into protective (less probable to cause autism) and risk (more probable to cause autism; Table 3). Risk alleles are listed as the following: in Chromosome 1: *IL24*-rs1150258C; rs1507765C (*value of p* = 2.3773 × 10^−5^); Chromosome 3: *PLCL2*-rs4602367A; *PLCL2*-rs17272796C (*value of p* = 3.2579 × 10^−5^); rs9854207; rs2642926 (*value of p* = 9.399 × 10^−6^); Chromosome 6: *OR5V1*-rs9257819A; *OR5V1*-rs2022077A; *OR12D2*-rs9257834G; *OR12D2*-rs4987411T; *OR12D2*-rs2073154C; *OR12D2*-rs2073153T; *OR12D2*-rs2073151G; *OR5V1*-rs2073149A; *OR5V1*-rs1028411T; *OR5V1*-rs2394607T (*value of p* = 4.5015 × 10^−5^); Chromosome 7: *AKAP9*-rs6964587T; *AKAP9*-rs6960867G; *AKAP9*-rs1063243C (*value of p* = 2.1723 × 10^−5^) and Chromosome 19; *ZNF600*-rs7507442G; *ZNF816*-rs57088011C (*value of p* = 7.3276 × 10^−5^; Table 3; Figure 2). Whereas the alleles of protective haplotypes are: in Chromosome 1: *IL24*-rs1150258T; rs1507765A (*value of p* = 5.9759 × 10^−5^); Chromosome 3: *PLCL2*-rs4602367G; *PLCL2*-rs272796T (*value of p* = 3.2579 × 10^−5^); rs9854207A; rs2642926C (*value of p* = 2 × 10^−4^); Chromosome 6: *OR5V1*-rs9257819C; *OR5V1*-rs2022077T; *OR12D2*-rs9257834T; *OR12D2*-rs4987411C; *OR12D2*-rs2073154G; *OR12D2*-rs2073153G; *OR12D2*-rs2073151A; *OR5V1*-rs2073149T; *OR5V1*-rs1028411G; *OR5V1*-rs2394607C (*value of p* = 1 × 10^−4^); Chromosome 7: *AKAP9*-rs6964587G; *AKAP9*-rs6960867A; *AKAP9*-rs1063243A (*value of p* = 6.272 × 10^−5^) and Chromosome 19; *ZNF600*-rs7507442A; *ZNF816*-rs57088011G (*value of p* = 2.8266 × 10^−5^; Tables 3; Figure 2). Surprisingly, olfactory receptor family 23 subfamily D member 2 (*OR12D2*) and olfactory receptor family 5 subfamily V member 1 (*OR5V1*) located on chromosome 6 had multiple significant nucleotide variants in Saudi autistic females (Figure 2).

**Table 3:** Haplotype blocks of SNPs with significant *p* < 0.0001 in Saudi autistic females.

Chr	Block	Haplotype	Freq.	Case, control ratio counts	Case, control frequencies	Chi square	Value of *p*	Haplotypes	Risk/protective
1	Block 1	CC	0.303	24.1: 19.9, 20.2: 81.8	0.548, 0.198	17.86	2.38 × 10^−05^	rs1150258C; rs1507765C	Risk
1		TA	0.303	3.1: 40.9, 41.2: 60.8	0.071, 0.404	16.11	5.98 × 10^−05^	rs1150258T; rs1507765A	Protective
1		TC	0.21	8.9: 35.1, 21.8: 80.2	0.202, 0.214	0.027	0.8688	rs1150258T; rs1507765C	
1		CA	0.183	7.9: 36.1, 18.8: 83.2	0.179, 0.185	0.006	0.9376	rs1150258C; rs1507765A	
3	Block 1	GT	0.657	18.0: 26.0, 78.0: 24.0	0.409, 0.765	17.261	3.26 × 10^−05^	rs4602367G; rs272796T	Protective
3		AC	0.329	25.0: 19.0, 23.0: 79.0	0.568, 0.225	16.361	5.24 × 10^−05^	rs4602367A; rs272796C	Risk
3	Block 2	AC	0.503	11.8: 32.2, 61.7: 40.3	0.269, 0.605	13.88	2.00 × 10^−04^	rs9854207A; rs2642926C	Protective
3		CT	0.325	25.8: 18.2, 21.7: 80.3	0.587, 0.212	19.63	9.40 × 10^−06^	rs9854207C; rs2642926T	Risk
3		AT	0.134	5.2: 38.8, 14.3: 87.7	0.118, 0.140	0.138	0.7106	rs9854207A; rs2642926T	
3		CC	0.038	1.2: 42.8, 4.3: 97.7	0.027, 0.042	0.207	0.6491	rs9854207C; rs2642926C	
6	Block 1	AAGTCTGATT	0.493	33.0: 11.0, 39.0: 63.0	0.750, 0.382	16.647	4.50 × 10^−05^	rs9257819A; rs2022077A; rs9257834G; rs4987411T; rs2073154C; rs2073153T; rs2073151G; rs2073149A; rs1028411T; rs2394607T	Risk
6		CTTCGGATGC	0.466	10.0: 34.0, 58.0: 44.0	0.227, 0.569	14.395	1.00 × 10^−04^	rs9257819C; rs2022077T; rs9257834T; rs4987411C; rs2073154G; rs2073153G; rs2073151A; rs2073149T; rs1028411G; rs2394607C	Protective
6		AAGTCTGTTC	0.027	1.0: 43.0, 3.0: 99.0	0.023, 0.029	0.052	0.8204	rs9257819A; rs2022077A; rs9257834G; rs4987411T; rs2073154C; rs2073153T; rs2073151G; rs2073149T; rs1028411T; rs2394607C	
6		AAGTCTGTTT	0.014	0.0: 44.0, 2.0: 100.0	0.000, 0.020	0.887	0.3463	rs9257819A; rs2022077A; rs9257834G; rs4987411T; rs2073154C; rs2073153T; rs2073151G; rs2073149T; rs1028411T; rs2394607T	
7	Block 1	GAA	0.589	15.0: 29.0, 71.0: 31.0	0.341, 0.696	16.019	6.27 × 10^−05^	rs6964587G; rs6960867A; rs1063243A	Protective
7		TGC	0.397	29.0: 15.0, 29.0: 73.0	0.659, 0.284	18.032	2.17 × 10^−05^	rs6964587T; rs6960867G; rs1063243C	Risk
19	Block 1	AG	0.514	11.0: 33.0, 64.0: 38.0	0.250, 0.627	17.531	2.83 × 10^−05^	rs7507442A; rs57088011G	Protective
19		GG	0.425	25.0: 19.0, 37.0: 65.0	0.568, 0.363	5.31	0.0212	rs7507442G; rs57088011G	
19		GC	0.062	8.0: 36.0, 1.0: 101.0	0.182, 0.010	15.724	7.33 × 10^−05^	rs7507442G; rs57088011C	Risk

Chr: chromosome; Freq.: frequency.

After conducting functional enrichment analysis of females’ gene list, genes with SNPs *p* < 0.00018 have shown a link to certain diseases including systemic lupus erythematosus disease (SLE; 5 Genes; *p* = 0.004400769; *BRD2*, *OR12D2*, *CR2*, *OR5V1*, *HLA-DOA*), Amyotrophic Lateral Sclerosis (3 Genes; *p* = 0.046868501; *CUBN*, *SIPA1L2*, *COMMD10*), as well as some pathways like regulation of complement cascade (2 Genes; *p* = 0. 0.0018; *CD55*, *CR2*) vitamin B12 metabolism (2 Genes; *p* = 0.006683; *CUBN, INSR*), and female preferences for male odors (2 Genes; *p* = 0.008301262; *OR12D2*, *OR5V1*). The most significant SNPs with *p* < 0.0001 (Supplementary Table 1) associated with autism in Saudi females were subjected for the functional annotation, there were 27 DAVID IDs. Gene ontology enrichment analysis indicated the significant (*p* value = 0.0051; involved 6 genes *MLXIPL*, *ZNF816*, *YEATS2*, *INSR*, *PROX2*, and *ZNF600*) biological process, regulation of DNA-templated transcription (GO:0006355).

## 4. Discussion

This study evaluates the risk of genetic variation in ASD Saudi female subjects. The most significant *SPHK1* SNP rs2247856 was reported recently as a significantly associated variant with Parkinson’s disease in both genders (19). GWAS catalog^2^ of rs2247856 reported the observed risk allele (rs2247856-A) of the present study with reticulocyte count, mean corpuscular volume, lymphocyte count, and reticulocyte fraction of red cells. However, no earlier reports on autism. No previous association was reported on rs386789496, rs6960867 and rs12035482. GWAS catalog of *PLCL2* SNP rs4602367-A reported the association with rheumatoid arthritis (20). Even though, *PLCL2* SNP rs4602367-A was not reported on autism, a recent study revealed the association of *PLCL2* SNPs (rs6800583 and rs73139272) with autism (21).

**Figure 3:**
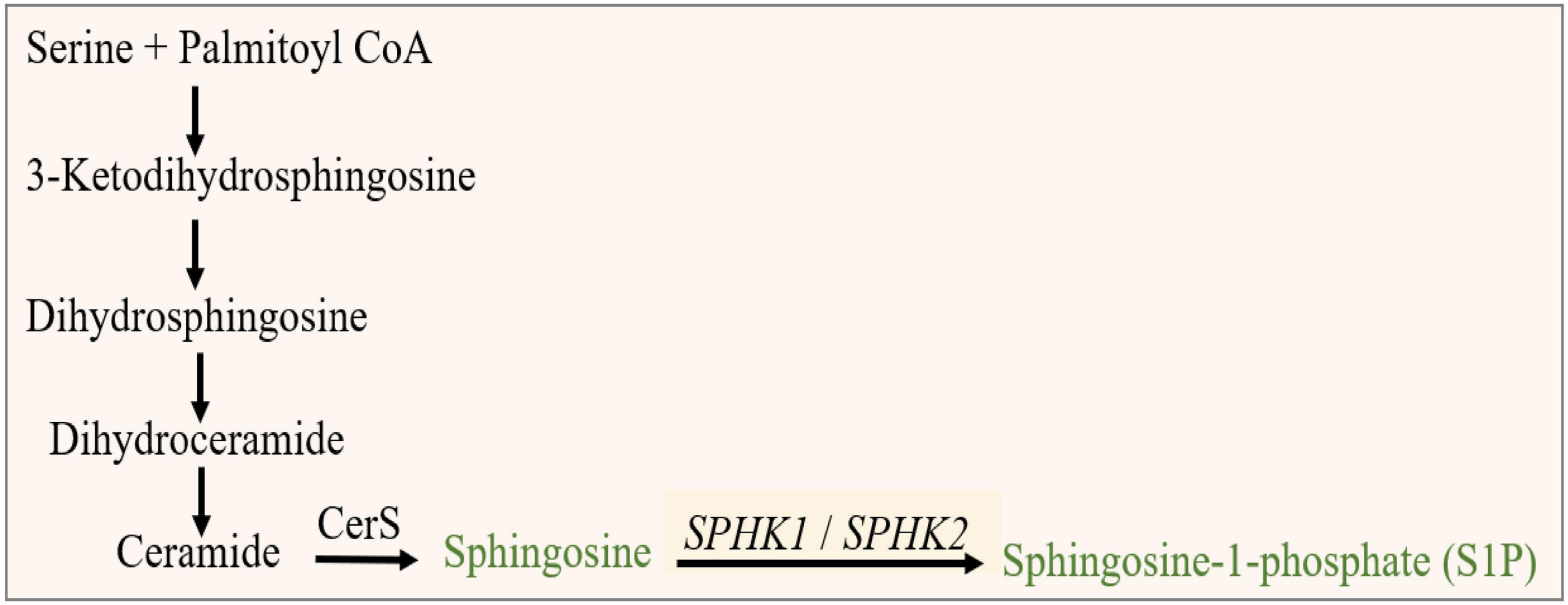
Sphingolipid metabolism pathway illustrates the processes of sphingosine-1-phosphate (S1P) production. The last step is catalyzed by *SPHK1*, a significant protein-coding gene associated with autism in Saudi females.

**Figure 4:**
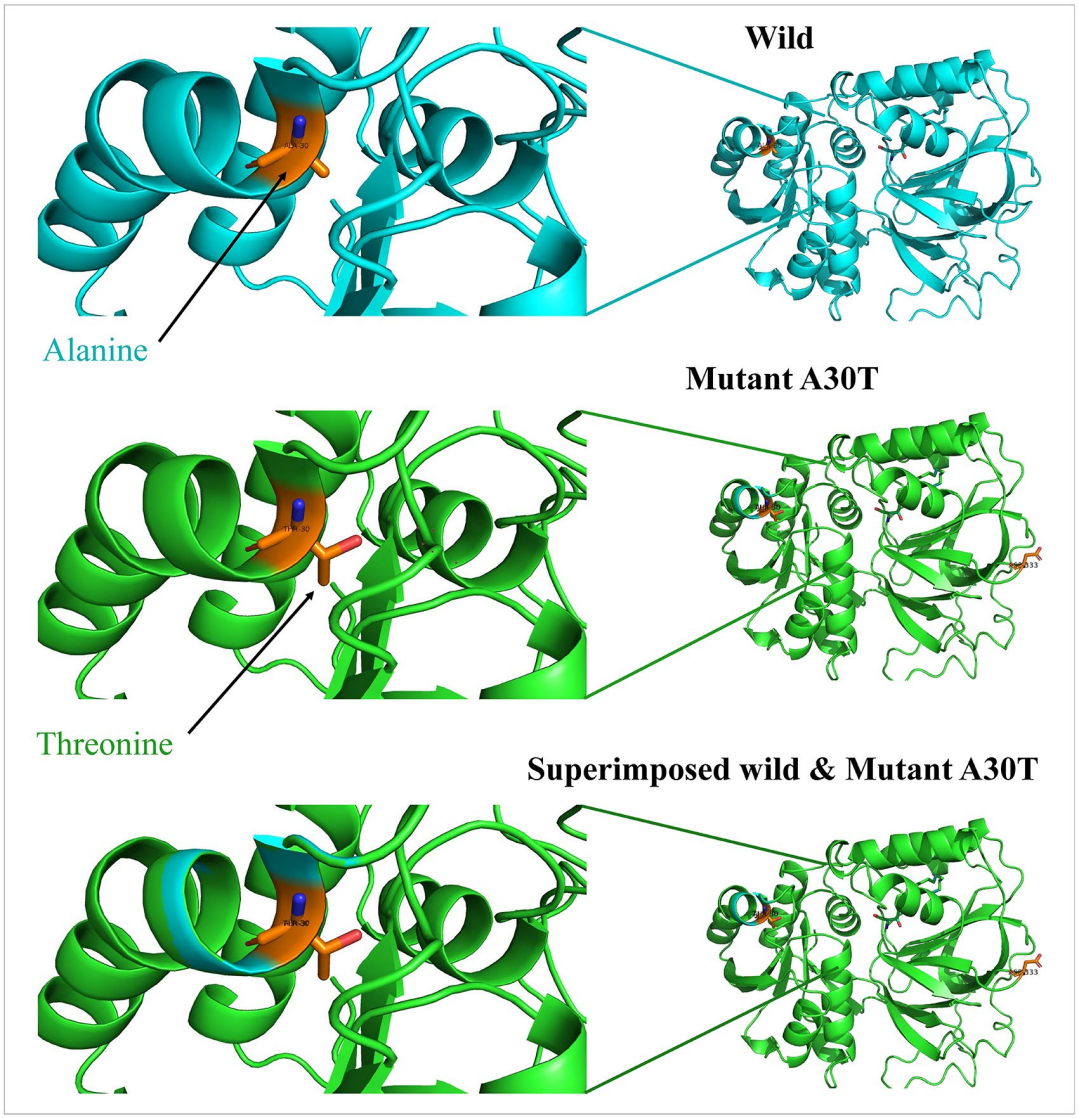
Protein models of *SPHK1* wild-type and mutated proteins. Root mean square deviation (RMSD) for the superimposed: 0.001.

Beginning with significant genes plotted in Manhattan plot, *SPHK1* has the highest *value of p* of 3.069 × 10^−6^. *SPHK1* is a key enzyme of sphingolipid metabolism which modulates cellular proliferation and pro-survival function (22). Since *SPHK1* and *SPHK2* phosphorylate sphingosine to sphingosine-1-phosphate (S1P) (23) (Figure 3), the presence of a high concentration of *SPHK1* increases the production of S1P which when elevated can lead to autism according to Wu et al. (24). In addition, based on multiple logistic regression analysis, S1P alterations were considered significant biomarker predictor for autism (23). Similarly, dysregulation of S1P triggers the manifestation of psychiatric and neurological diseases such as Alzheimer’s disease (25), schizophrenia (26) Parkinson’s disease (27) and anxiety disorder (28). However, an experiment done on valproic acid rat model found that protein expression of *SPHK1* wasn’t significant as it did not reach the significance level (24). Protein modeling of mutated SPHK1 denotes the damaging changes, which indicates the mutated SPHK1 protein can affect the Sphingolipid metabolism pathway (Figure 4). Some findings reported proteins encoded by *AKAP9, another significant gene in the allelic association study*, to be highly expressed in autism subjects (29). *Yet, the mechanism of the association is still unknown*.

**Figure 5:**
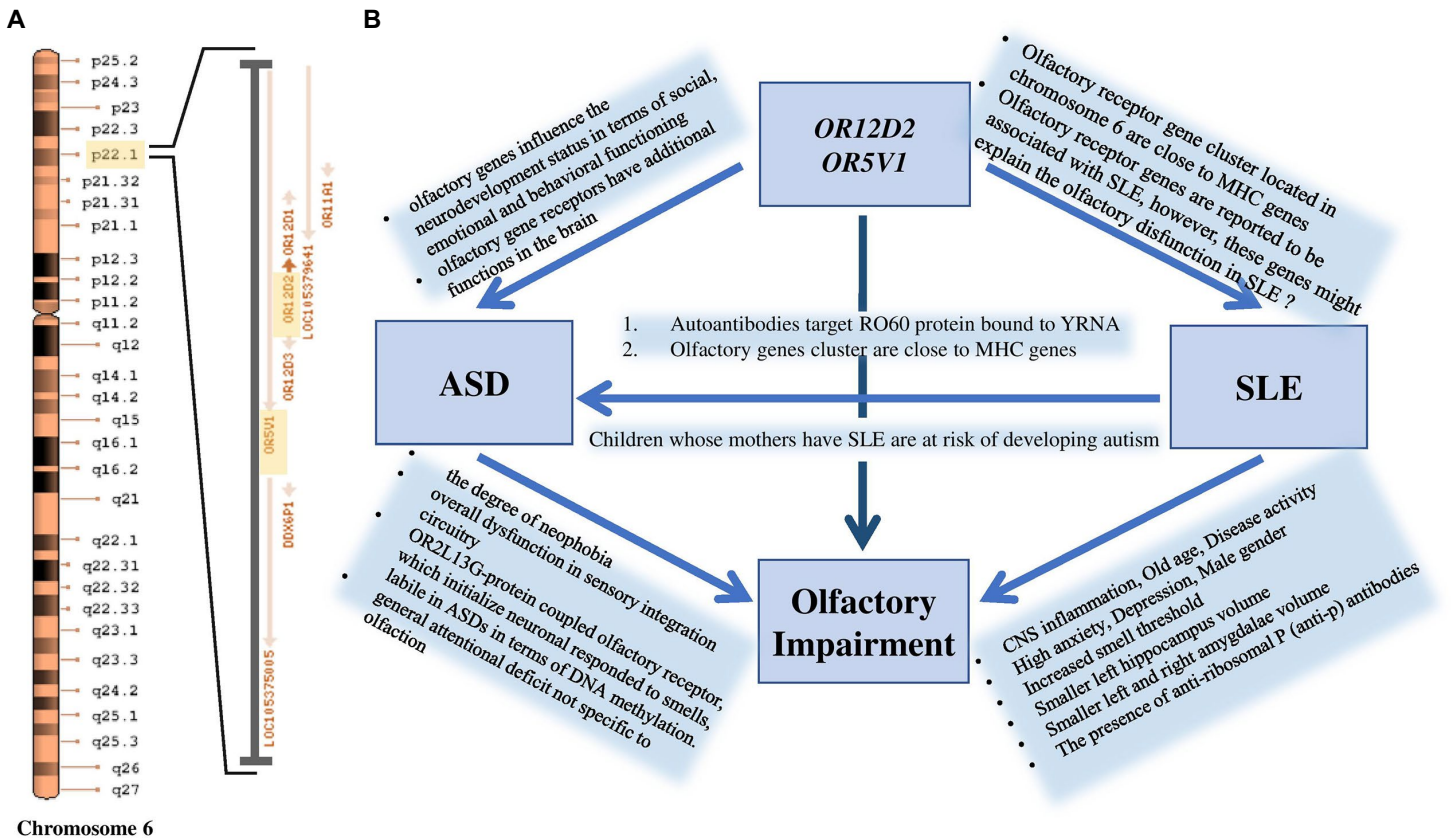
**(A)** Significant ASD variation at *OR12D2* and *OR5V1* genes are located within a cluster of olfactory receptor genes at the chromosomal region 6p22.1. **(B)** The figure shows that maternal SLE influences the manifestation of ASD in their offspring. Smell deficits and variations in olfactory receptor genes, particularly in *OR12D2* and *OR5V1*, are common in both ASD and SLE patients.

Significant SNP candidates for ASD etiology in females were perceived to be located at *OR12D2* and *OR5V1* genes on chromosome 6 having high linkage disequilibrium (Figure 2). Several studies have perceived sensory abnormalities in autism subjects including unusual odor perception (30, 31). Indeed, olfactory genes influence the neurodevelopment status in terms of social, emotional and behavioral functioning (30, 32). A study reported a link between a cluster of SNPs located within the olfactory receptor genes on chromosome 6p22.1 and social defects in ASD ( (33); Figure 5). Interestingly, Systemic Lupus Erythematosus (SLE), which is an autoimmune disease closely related to autism, is accompanied by variations in olfactory receptor genes (Figure 5). A research conducted in Egypt revealed that 7 out of 38 autoimmune ASD patients had a family history of SLE (34). The largest cohort study done on 719 SLE offspring reported a strong association between the two disorders (35). Further evidence supporting the relationship between the two disorders is found through the functional enrichment analysis suggesting that *OR12D2* and *OR5V1* are commonly affected genes in both SLE and female ASD patients. Large cluster of olfactory receptor genes on chromosome 6 is located in proximity to class 1 histocompatibility complex genes which mediate immunity (36). Another factor that attributes to the development of ASD in SLE’s offspring is the presence of the autoimmune antibodies in patients with SLE which attack the Ro60 protein bound to YRNA (37, 38). All these factors justify the reason behind the doubled risk of ASD in SLE patients’ offspring, giving that 21.4–26% of SLE offspring have autism (39). Current studies have emphasized on the potential role for the immune system in ASD, with immune-genetic abnormalities and the inappropriate response of the immune system to environmental challenges. A meta-analysis of 7 observational studies (25,005 ASD cases and 4,543,321 participants) was conducted assessing the relationships between maternal systemic lupus erythematosus (SLE) or rheumatoid arthritis (RA) and risk for ASD in offspring. The results showed that maternal RA was associated with an increased risk for ASDs, whereas maternal SLE was associated with an increased risk for ASD only in western population (40, 41).

Another medical condition that shares common genes with ASD is vitamin B12 (cobalamin) deficiency, which causes many neurological and psychiatric disorders. Cobalamin catalyzes the conversion of homocysteine to methionine (1, 42). A study conducted in Oman on 80 participants half of which are cases revealed an accumulation of homocysteine and reduced levels of methionine due to vitamin B12 insufficiency (43). Another study attributed the association between Vitamin B12 and autism to the role of vitamin B12 in the methylation cycle and genetic material biosynthesis (44). Biochemical abnormalities related with ASD consist of impaired methylation and sulphation capacities beside low glutathione (GSH) redox capacity. Possible managements for these abnormalities comprise cobalamin (B12). A systematic review of a total 17 studies was identified studies using vitamin B12 to manage ASD. The study found that generally; vitamin B12 seems to have evidence for efficacy in patients with ASD, especially in individuals who have been identified with unfavorable biochemical profiles. Initial clinical evidence proposes that vitamin B12, mostly subcutaneously injected, improves metabolic abnormalities in ASD alongside with clinical symptoms. Cobalamin is a promising supplement used in the management of ASD (45). The limitations of the current study should be acknowledged. First, the pilot study design nature and its relatively small sample size.

## 5. Conclusion

In summary, the findings of this study provide the first evidence for female-based genetic analysis in Saudi Arabia and assess the relationship between olfactory receptor genes and ASD. Furthermore, variations on olfactory receptor genes elucidate the impact of SLE in females and the inheritance of ASD. Future investigations with more representative samples that include experiments on rat models are needed to practically prove the association and enhance ASD managing choices.

## Data availability statement

The datasets presented in this study can be found in online repositories. The names of the repository/repositories and accession number(s) can be found at: GEO database, under accession GSE221098.

## Ethics statement

The studies involving human participants were reviewed and approved by Institutional Review Board (IRB) of Imam Abdulrahman Bin Faisal University (IRB-2016-13-152). Written informed consent to participate in this study was provided by the participants' legal guardian/next of kin.

## Author contributions

NA, AA, SA, JB: conceptualization, data curation, and investigation. MA, HA, SA, JB, and NA: formal analysis. NA: funding acquisition. NA, SA, and JB: methodology, project administration, resources, software, supervision, validation, and visualization. MA, HA, AA, SA, JB, and NA: writing—original draft. All authors contributed to the article and approved the submitted version.

## Funding

This work was supported by the Deanship of Scientific Research, Imam Abdulrahman Bin Faisal University (Grant No: 2016-057-IRMC).

## Conflict of interest

The authors declare that the research was conducted in the absence of any commercial or financial relationships that could be construed as a potential conflict of interest.

## Publisher’s note

All claims expressed in this article are solely those of the authors and do not necessarily represent those of their affiliated organizations, or those of the publisher, the editors and the reviewers. Any product that may be evaluated in this article, or claim that may be made by its manufacturer, is not guaranteed or endorsed by the publisher.

## Footnotes

^1^ https://www.ncbi.nlm.nih.gov/geo/query/acc.cgi?acc=GSE221098

^2^ https://www.ebi.ac.uk/gwas/variants/rs2247856
