# Supplementary material for: Exome-wide analysis identify multiple variations in olfactory receptor genes (OR12D2 and OR5V1) associated with autism spectrum disorder in Saudi females
Source: Front Med (Lausanne). 2023 Feb 1;10:1051039. doi: 10.3389/fmed.2023.1051039 (PMC9928728; doi:10.3389/fmed.2023.1051039)
Supplement: Supplementary file 1 [file Table_1.docx]

**Exome-wide analysis identify multiple variations in olfactory receptor genes (*OR12D2* and *OR5V1*) associated with autism spectrum disorder in Saudi females**

Noor B. Almandil, Maram Adnan Alismail, Hind Saleh Alsuwat, Abdulla AlSulaiman, Sayed AbdulAzeez, and J. Francis Borgio

**Supplementary Table 1.** The most significant SNPs with p <0.0001 associated with autism in Saudi females.

| **S.NO** | **CHR** | **SNP ID** | **BP** | **MA** | **MAF** | **Gene** | **AA** | ***P* value** | | **CHISQ** | | **OR(L95-U95)** | | **Case, Control Frequencies** | **HWpval** | |
| --- | --- | --- | --- | --- | --- | --- | --- | --- | --- | --- | --- | --- | --- | --- | --- | --- |
| 1 | 17 | [rs2247856](http://www.ncbi.nlm.nih.gov/SNP/snp_ref.cgi?rs=2247856) | 74381555 | A | 0.247 | *SPHK1* | A | 3.07×10^-06^ | | 21.77 | | 6.286(2.777-14.23) | | 0.500, 0.137 | 0.0017 | |
| 2 | 16 | rs386789496 | 17988303 | A | 0.473 | *LOC107984893* | A | 1.04×10^-05^ | | 19.44 | | 5.5(2.486-12.17) | | 0.750, 0.353 | 0.0117 | |
| 3 | 3 | rs4602367 | 17053499 | A | 0.336 | *PLCL2* | A | 1.78×10^-05^ | | 18.41 | | 4.961(2.321-10.6) | | 0.591, 0.225 | 0.2088 | |
| 4 | 7 | rs6960867 | 91712698 | G | 0.397 | *AKAP9* | G | 2.17×10^-05^ | | 18.03 | | 4.867(2.282-10.38) | | 0.659, 0.284 | 0.588 | |
| 5 | 1 | rs12035482 | 195738953 | A | 0.493 | none | G | 2.32×10^-05^ | | 17.91 | | 0.188(0.08352-0.4234) | | 0.773, 0.390 | 0.0717 | |
| 6 | 19 | rs7507442 | 53278953 | G | 0.486 | *ZNF600* | G | 2.83×10^-05^ | | 17.53 | | 5.053(2.289-11.15) | | 0.750, 0.373 | 0.0396 | |
| 7 | 7 | rs6964587 | 91630620 | A | 0.403 | *AKAP9* | T | 3.43×10^-05^ | | 17.17 | | 4.8(2.222-10.37) | | 0.667, 0.294 | 0.3397 | |
| 8 | 5 | rs160632 | 96503523 | G | 0.445 | *RIOK2* | C | 3.46×10^-05^ | | 17.15 | | 4.769(2.214-10.27) | | 0.705, 0.333 | 0.1332 | |
| 9 | 3 | rs9854207 | 27614316 | C | 0.363 | none | C | 3.53×10^-05^ | | 17.11 | | 4.643(2.187-9.855) | | 0.614, 0.255 | 0.1288 | |
| 10 | 19 | rs142920057 | 334472 | C | 0.121 | *MIER2* | G | 4.29×10^-05^ | | 16.74 | | 8.143(2.643-25.09) | | 0.300, 0.050 | 0.6628 | |
| 11 | 6 | rs2073149 | 29365423 | A | 0.493 | *OR12D2* | A | 4.30×10^-05^ | | 16.74 | | 4.895(2.215-10.82) | | 0.750, 0.380 | 0.3153 | |
| 12 | 4 | rs1339 | 154631563 | G | 0.197 | *RNF175* | C | 5.60×10^-05^ | | 16.23 | | 5.502(2.285-13.25) | | 0.405, 0.110 | 0.5161 | |
| 13 | 7 | rs10488360 | 4411209 | A | 0.452 | none | A | 5.67×10^-05^ | | 16.21 | | 4.565(2.122-9.818) | | 0.705, 0.343 | 0.4184 | |
| 14 | 5 | rs409045 | 34628627 | G | 0.37 | none | C | 6.14×10^-05^ | | 16.06 | | 4.412(2.085-9.335) | | 0.614, 0.265 | 0.4098 | |
| 15 | 7 | rs1063243 | 91726927 | C | 0.411 | *AKAP9* | C | 6.27×10^-05^ | | 16.02 | | 4.428(2.086-9.399) | | 0.659, 0.304 | 0.5296 | |
| 16 | 19 | rs57088011 | 53454387 | G | 0.062 | *ZNF816* | C | 7.33×10^-05^ | | 15.72 | | 22.44(2.712-185.8) | | 0.182, 0.010 | 0.4609 | |
| 17 | 5 | rs11556045 | 73985215 | G | 0.233 | *HEXB* | A | 7.95×10^-05^ | | 15.57 | | 0.04863(0.006415-0.3686) | | 0.977, 0.676 | 0.282 | |
| 18 | 1 | rs669408 | 232519150 | C | 0.35 | none | C | 8.77×10^-05^ | | 15.38 | | 4.5(2.067-9.795) | | 0.600, 0.250 | 1 | |
| 19 | 3 | rs2642926 | 27615419 | A | 0.459 | none | T | 9.15×10^-05^ | | 15.3 | | 4.372(2.036-9.389) | | 0.705, 0.353 | 0.0125 | |
| 20 | 19 | rs7248104 | 7224431 | A | 0.459 | *INSR* | A | 9.15×10^-05^ | | 15.3 | | 4.372(2.036-9.389) | | 0.705, 0.353 | 0.9049 | |
| 21 | 6 | rs2073153 | 29364835 | C | 0.472 | *OR12D2* | T | 9.17×10^-05^ | | 15.3 | | 0.2116(0.09399-0.4762) | | 0.773, 0.418 | 0.3848 | |
| 22 | 3 | rs17272796 | 17077268 | G | 0.336 | *PLCL2* | C | 9.29×10^-05^ | | 15.28 | | 4.276(2.016-9.069) | | 0.568, 0.235 | 0.2088 | |
| 23 | 7 | rs10260011 | 84709356 | A | 0.226 | *SEMA3D* | T | 9.44×10^-05^ | | 15.25 | | 4.777(2.102-10.86) | | 0.432, 0.137 | 0.5474 | |
| 24 | 1 | rs41268336 | 16069525 | A | 0.075 | *TMEM82* | T | 0.0001024 | 15.09 | | 12.86(2.649-62.41) | | 0.205, 0.020 | | 0.0863 |  |
| 25 | 17 | rs62637603 | 8215927 | A | 0.075 | *ARHGEF15* | T | 0.0001024 | 15.09 | | 12.86(2.649-62.41) | | 0.205, 0.020 | | 0.6678 |  |
| 26 | 6 | rs2394607 | 29369519 | G | 0.493 | *OR5V1* | T | 0.0001135 | 14.9 | | 0.224(0.1018-0.4931) | | 0.750, 0.402 | | 0.6822 |  |
| 27 | 5 | rs1129495 | 115428381 | C | 0.103 | *COMMD10* | G | 0.0001185 | 14.82 | | 8.167(2.434-27.4) | | 0.250, 0.039 | | 1 |  |
| 28 | 12 | rs1129649 | 6948468 | G | 0.445 | *P3H3* | T | 0.0001216 | 14.77 | | 0.2112(0.09212-0.4843) | | 0.795, 0.451 | | 0.0477 |  |
| 29 | 7 | rs13235516 | 130923681 | G | 0.315 | *MKLN1* | T | 0.0001284 | 14.66 | | 0.1429(0.04751-0.4295) | | 0.909, 0.588 | | 0.4545 |  |
| 30 | 1 | rs1150258 | 207074905 | G | 0.486 | *IL24* | G | 0.0001302 | 14.64 | | 4.308(1.986-9.344) | | 0.727, 0.382 | | 1 |  |
| 31 | 1 | rs12137794 | 6705944 | A | 0.041 | *DNAJC11* | T | 0.0001398 | 14.51 | | 0.0289(0.0016 -0.5252) | | 0.136, 0.000 | | 1 |  |
| 32 | 3 | rs74496163 | 183508714 | A | 0.041 | *YEATS2* | A | 0.0001398 | 14.51 | | 34.6104(1.9039 -629.1744) | | 0.136, 0.000 | | 1 |  |
| 33 | 7 | rs202093717 | 73011550 | A | 0.041 | *MLXIPL* | T | 0.0001398 | 14.51 | | 0.0109(0.0006 -0.2035) | | 0.136, 0.000 | | 1 |  |
| 34 | 1 | rs4846066 | 11938050 | A | 0.465 | none | G | 0.0001433 | 14.46 | | 0.2206(0.09806-0.4962) | | 0.773, 0.429 | | 1 |  |
| 35 | 14 | rs8014204 | 75322794 | G | 0.322 | *PROX2* | G | 0.0001465 | 14.42 | | 4.122(1.94-8.756) | | 0.545, 0.225 | | 0.0072 |  |
| 36 | 6 | rs9257819 | 29360183 | C | 0.466 | *OR5V1* | A | 0.0001482 | 14.4 | | 0.2231(0.09959-0.4999) | | 0.773, 0.431 | | 0.3984 |  |
| 37 | 6 | rs2022077 | 29361124 | T | 0.466 | *OR5V1* | A | 0.0001482 | 14.4 | | 0.2231(0.09959-0.4999) | | 0.773, 0.431 | | 0.3984 |  |
| 38 | 6 | rs9257834 | 29364615 | A | 0.466 | *OR12D2* | G | 0.0001482 | 14.4 | | 0.2231(0.09959-0.4999) | | 0.773, 0.431 | | 0.3984 |  |
| 39 | 6 | rs4987411 | 29364643 | G | 0.466 | *OR12D2* | T | 0.0001482 | 14.4 | | 0.2231(0.09959-0.4999) | | 0.773, 0.431 | | 0.3984 |  |
| 40 | 6 | rs2073154 | 29364815 | G | 0.466 | *OR12D2* | C | 0.0001482 | 14.4 | | 0.2231(0.09959-0.4999) | | 0.773, 0.431 | | 0.3984 |  |
| 41 | 6 | rs2073151 | 29364951 | A | 0.466 | *OR12D2* | G | 0.0001482 | 14.4 | | 0.2231(0.09959-0.4999) | | 0.773, 0.431 | | 0.3984 |  |
| 42 | 6 | rs1028411 | 29367399 | C | 0.466 | *OR5V1* | T | 0.0001482 | 14.4 | | 0.2231(0.09959-0.4999) | | 0.773, 0.431 | | 0.3984 |  |
| 43 | 6 | rs2281438 | 150525260 | G | 0.445 | *PPP1R14C* | C | 0.0001579 | 14.28 | | 4.102(1.929-8.725) | | 0.682, 0.343 | | 0.1332 |  |
| 44 | 10 | rs3740168 | 16948390 | C | 0.042 | *CUBN* | C | 0.0001618 | 14.23 | | 18.75(3.9836 -88.2517) | | 0.136, 0.000 | | 1 |  |
| 45 | 1 | rs10798302 | 173987798 | G | 0.281 | none | A | 0.0001733 | 14.1 | | 0.1232(0.0357-0.4255) | | 0.932, 0.627 | | 0.6126 |  |
| 46 | 19 | rs306481 | 56487603 | A | 0.384 | *NLRP8* | *A* | 0.0001733 | 14.1 | | 3.998(1.9-8.412) | | 0.614, 0.284 | | 0.6597 |  |
| 47 | 1 | rs1507765 | 207535246 | A | 0.486 | none | C | 0.0001754 | 14.08 | | 0.2333(0.1061-0.5132) | | 0.750, 0.412 | | 0.2667 |  |
| 48 | 6 | rs206767 | 32962420 | A | 0.471 | none | A | 0.0001834 | 13.99 | | 4.156(1.925-8.972) | | 0.705, 0.365 | | 0.6073 |  |
| 49 | 10 | rs1592051 | 93542186 | A | 0.438 | *TNKS2-AS1* | *C* | 0.0001843 | 13.98 | | 0.2197(0.09586-0.5037) | | 0.795, 0.461 | | 0.4462 |  |

S.NO: Serial number; CHR: Chromosome; SNP ID: Single nucleotide polymorphism ID; BP: Base pair position at the respective chromosome as per GRCh37.p13; MA: Minor allele name; MAF: Frequency of minor allele in controls; AA: Associated Allele; P: p-value; ChisQ: Basic allelic test chi-square; P: p-value; OR: Odd ratio; L95: Lower bound of 95% confidence interval for odds ratio; U95: Upper bound of 95% confidence interval for odds ratio.; CCF: Case, Control Frequencies; HWpval: p-value of Hardy-Weinberg equilibrium.
